# Case Report: Durable complete pathologic response and organ preservation following ipilimumab and nivolumab for locally advanced primary vaginal mucosal melanoma

**DOI:** 10.3389/fonc.2022.1044587

**Published:** 2022-11-30

**Authors:** Ahmad A. Tarhini, Wissam B. Hanayneh, John J. Powers, Carlos M. Moran Segura, Jose R. Conejo-Garcia, Cesar A. Lam, Ardeshir Hakam, Mitchel S. Hoffman

**Affiliations:** H. Lee Moffitt Cancer Center and Research Institute, University of South Florida, Tampa, FL, United States

**Keywords:** case report, vaginal mucosal melanoma, ipilimumab, nivolumab, immunotherapy

## Abstract

Optimal management of locally advanced vaginal mucosal melanoma is poorly understood because of its rarity and unique biology. Patients have a poor prognosis despite aggressive management approaches including pelvic exenteration and adjuvant radiation that carry major morbidities. We report a case of a patient in early 40’s who experienced complete pathologic response and organ preservation following immunotherapy consisting of 3 cycles of ipilimumab and nivolumab. Treatment was complicated by a high-grade immune mediated hepatitis that eventually resolved with immunosuppressive therapy. Immune monitoring studies utilizing vaginal tumor biopsies showed evidence of enhanced infiltration by CD3+/CD8+ cytotoxic T-cells and increased expression of MHC-I/PD-L1 within the tumor microenvironment following immunotherapy. The patient continues to be without evidence of disease recurrence by radiologic and gynecologic examinations with more than 2 years of follow up from the time of immunotherapy initiation. To our knowledge, this is the only case report in the literature of a patient with locally advanced vaginal mucosal melanoma experiencing a durable complete pathologic response and organ preservation following immune checkpoint blockade as the only treatment approach.

## Introduction

Mucosal melanoma arising from melanocytes of the female genital tract is rare and occurs primarily in the vulva, followed by the vagina (1). It carries a worse prognosis compared to cutaneous melanoma and has unique biological and molecular characteristics, including less frequent BRAF and a higher incidence of KIT and NRAS mutations (1). Genitourinary mucosal melanomas are more common in females than males, occurring at 1.8 cases per million women per year in the U.S. alone (2). Vaginal melanomas make up around 27% of genitourinary melanomas and 0.2% of all melanomas. Treatment for localized vulvovaginal melanoma is typically surgical and often associated with significant morbidity and organ loss, while 5-year overall survival rates are poor, ranging from 5 to 30% (3–5). Immune checkpoint inhibitors have changed the landscape of management of advanced cutaneous melanoma; however, their use in mucosal melanomas has been limited to the treatment of metastatic disease with no reported experiences in the neoadjuvant setting (6). In this report, we present a case of a pre-menopausal woman with a locally advanced primary vaginal melanoma definitively treated with front-line combination immunotherapy achieving a durable complete pathologic response and organ preservation. To our knowledge, there are no other similar reports in the literature.

## Case

**Figure 1:**
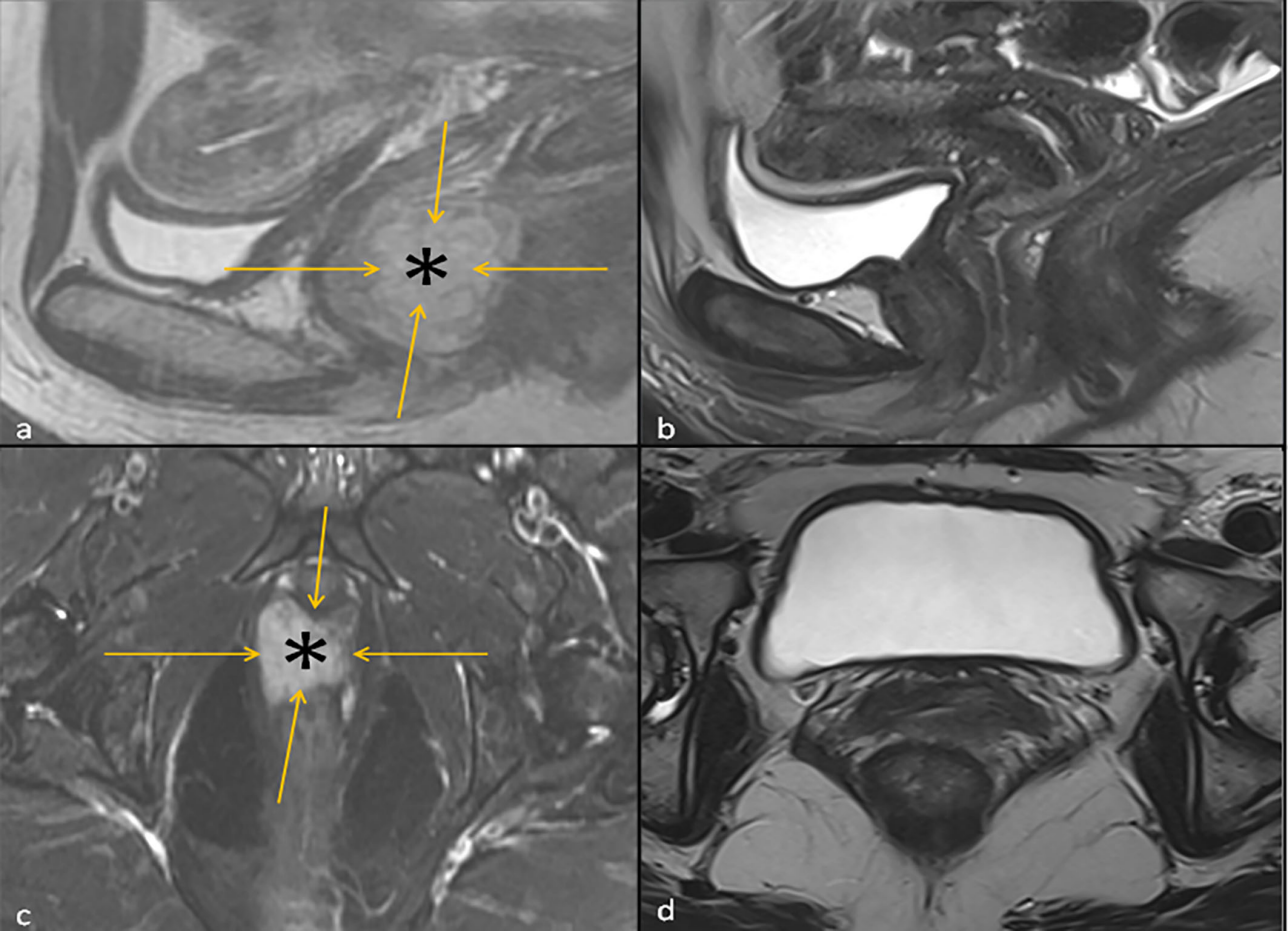
Left: T2 Sagittal Image **(A)** and fat suppressed T2 Axial Image **(C)** of the Pelvis at baseline showing a hyperintense vaginal mass (asterisk) outlined by orange arrows. Right: T2 Sagittal Image of the Pelvis **(B)** and Axial Image of the Pelvis **(D)** 3 months following the initiation ipilimumab and nivolumab neoadjuvant therapy, where the hyperintense vaginal mass is no longer visualized.

**Figure 2:**
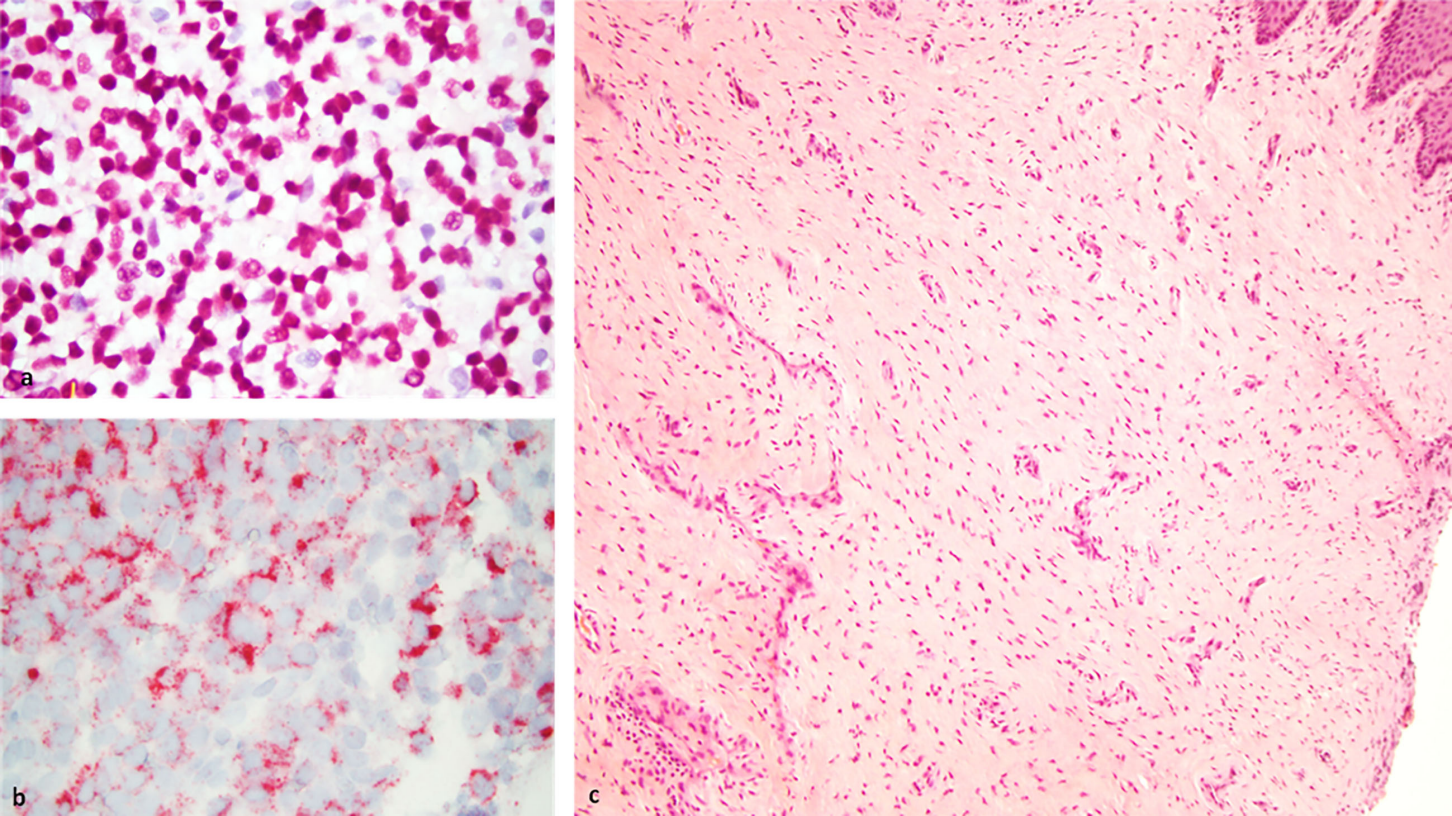
Left: Histology slides at baseline showing neoplastic cells using SOX10 **(A)** and HMB45 **(B)** immunostains at 63X magnification. Right: Biopsy of tumor site displaying complete resolution of the tumor with no viable tumor cells and scar tissue formation as assessed 3 months following ipilimumab and nivolumab neoadjuvant therapy **(C)**.

A patient in early 40’s presented to the gynecology clinic complaining of a vaginal mass that protruded with straining. The patient was an athletic and healthy individual with no significant medical history and no history of tobacco use or regular alcohol use. The patient had no family history of first-degree family members with malignancies. A gynecologic rectovaginal examination revealed a polypoid lesion likely extending from the posterior vaginal wall to the right parametrial tissue and anteriorly extending up to the urethra. Magnetic resonance imaging (MRI) of the pelvis revealed a large (3.7 x 2.0 x 2.6 cm) right lateral vaginal wall mass with apparent right parametrial extension and possible involvement of the anterior rectal wall, **Figure 1**. Systemic imaging with positron emission tomography showed a fluorodeoxyglucose-avid mass in the vagina with standardized uptake values up to 7.8 without any other uptake sites, **Figure 1**. A biopsy was obtained, and pathology revealed a malignant mucosal melanoma, **Figure 2**. The neoplastic cells showed immunoreactivity for SOX10, HMB45, and PAX8. They were negative for pan-cytokeratin, actin, CD10 and desmin. Next-generation sequencing of the tumor cells revealed an NRAS p.G12D mutation, but no mutations were noted in BRAF or KIT genes.

The patient was evaluated by a multidisciplinary oncology team and offered the radical surgical route of posterior pelvic exenteration but declined and requested less aggressive management. The patient’s case was discussed during a multidisciplinary tumor board. Considering the patient’s preference, the tumor board recommended neoadjuvant systemic immunotherapy with the combination of ipilimumab and nivolumab. The plan was presented to the patient who also agreed to have an interim assessment at 6 weeks following the initiation of immunotherapy and potentially reconsider the originally recommended surgical approach if no response was seen. Neoadjuvant therapy consisted of ipilimumab 3 mg/kg in combination with nivolumab 1 mg/kg every 3 weeks for 2 doses, followed by a gynecologic examination and radiologic imaging of the pelvis at 6 weeks. At 6 weeks, the patient was evaluated by gynecology oncology and completed a CT scan of the pelvis. Gynecologic evaluation indicated approximately 50% reduction in tumor size that was consistent with the findings on pelvic CT. Upon further discussion, agreement was made to proceed with 2 additional cycles of combination immunotherapy. However, following the 3^rd^ cycle, treatment was discontinued due to the development of grade 3 immune-mediated hepatitis. High-dose corticosteroids were initiated with initial limited response leading to the addition of mycophenolate mofetil and a taper schedule over about 4 months with eventual complete resolution of the hepatitis.

In the interim, a repeat MRI 3 months after the initiation of immunotherapy showed continued regression of the vaginal mass down to 2.6 x 0.8 cm. The patient underwent 12 circumferential vaginal biopsies shortly after the MRI, and all 12 biopsies showed no evidence of viable melanoma tumor cells, indicating a complete pathologic response (pCR) as illustrated in **Figure 2**. At 6 months following immunotherapy initiation, an MRI showed a radiologic complete response with complete resolution of the mass. This status persisted as imaged by PET/CT, transvaginal ultrasound and MRI imaging with continued follow-up up to 2 years; **Figure 1**.

**Figure 3:**
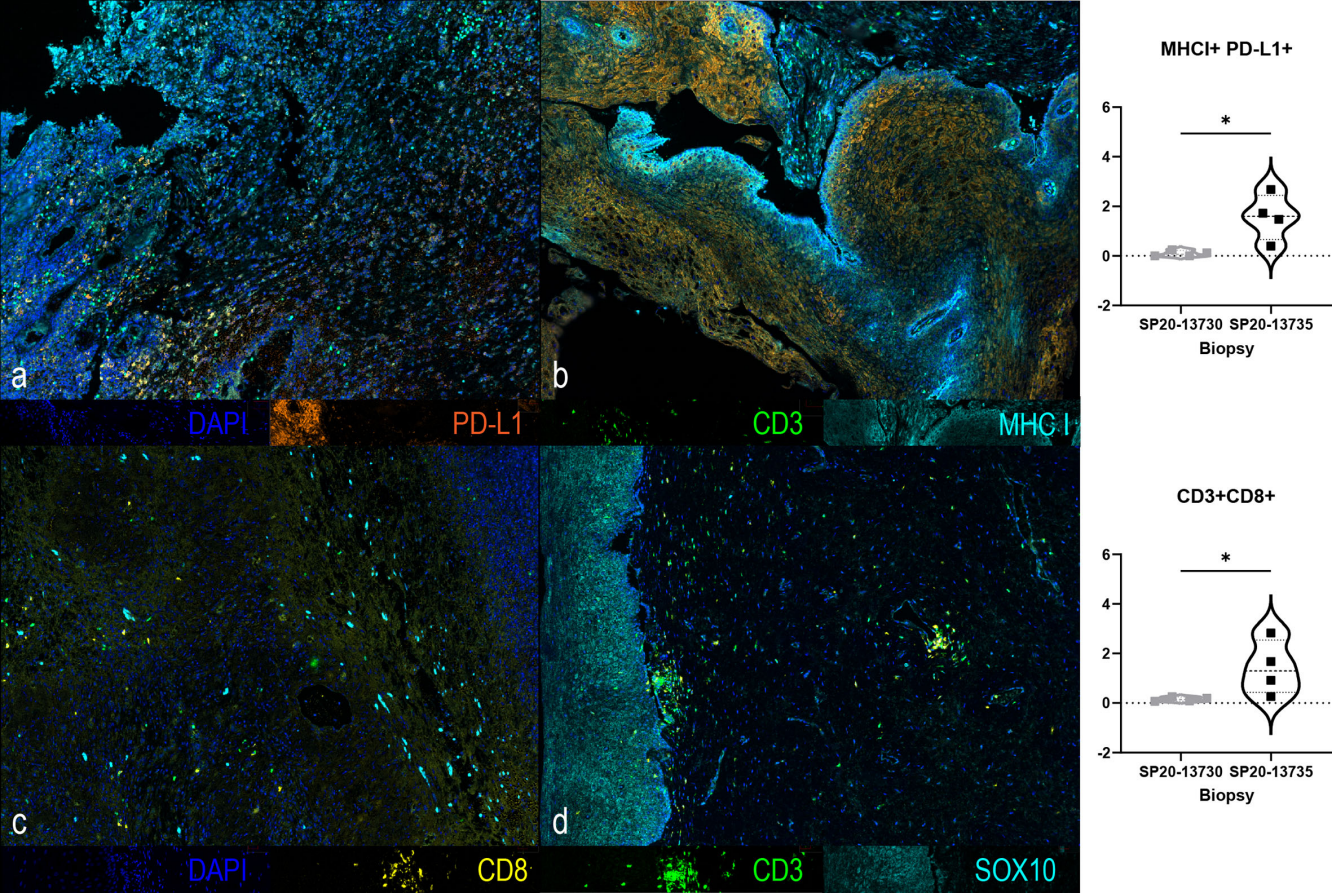
Multiplex immunofluorescence immunohistochemistry of tumor biopsies at baseline **(A, C)** and at 3 months **(B, D)** following the initiation of ipilimumab and nivolumab showing changes in CD3 (green), CD8 (yellow), PD-L1 (orange), MHCI (cyan), SOX10 (cyan) and DAPI (blue) immunostains. A significant increase in the expression of MHC-I+/PD-L1+ (p=0.0286, 2-Tailed Mann-Whitney) and of CD3+/CD8+ (p=0.0286, 2-Tailed Mann-Whitney) was found at 3 months compared to baseline. *, It designates a p value lower than 0.05.

**Figure 4:**
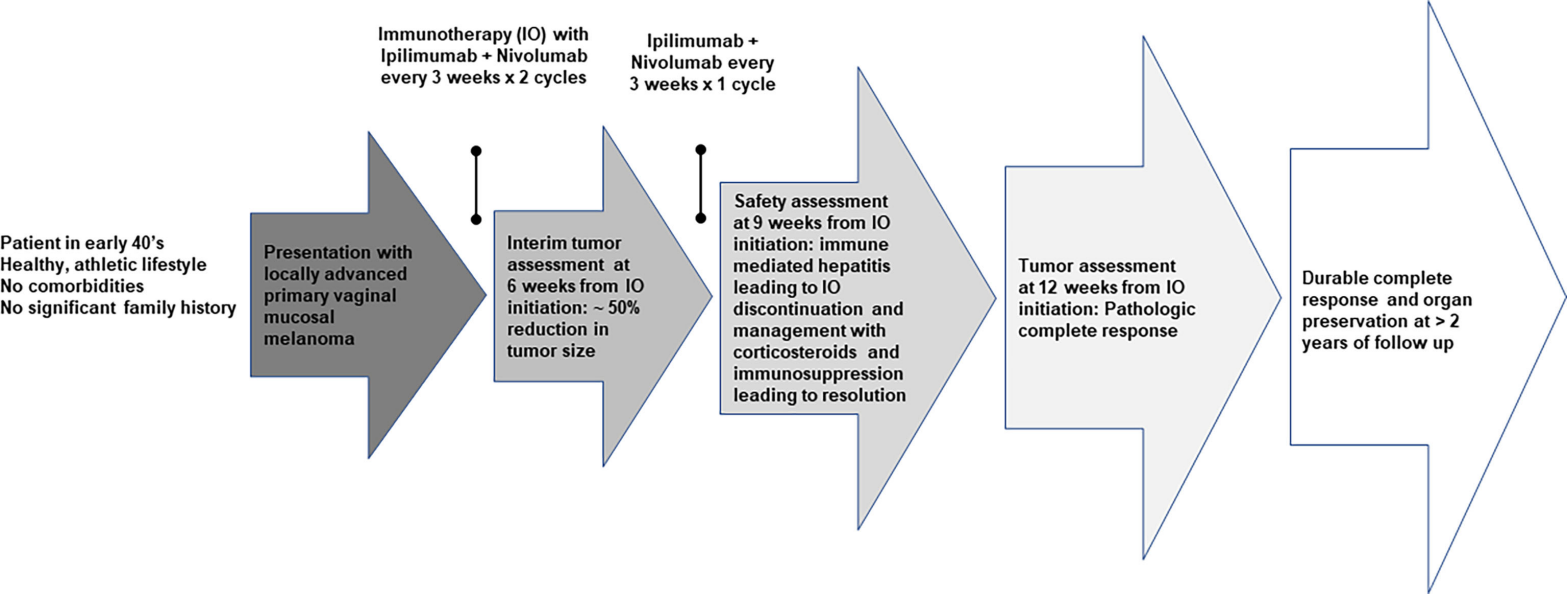
Timeline with relevant data from the diagnosis.

We conducted immune monitoring studies utilizing vaginal tumor biopsies, at baseline and 3 months following the initiation of immunotherapy using multiplex immunofluorescence immunohistochemistry (IHC) as previously described (7). Our analysis revealed significant increase in CD3+ CD8+ T-cell infiltration (*P* = 0.03) associated with enhanced PD-L1/MHC-I expression (*P* = 0.03) following immunotherapy. IHC results are presented in **Figure 3**. A timeline with relevant data from the time of diagnosis is summarized in **Figure 4**.

## Discussion

Vaginal melanoma more commonly affects postmenopausal women in their sixth and seventh decades but has been reported in lower age groups (2). It occurs more commonly in the lower third of the vagina and on the anterior vaginal wall compared with other sites. Most of these tumors are pigmented, although rare cases of amelanotic melanoma have been reported (5, 8). The International Federation of Gynecology and Obstetrics (FIGO) system for vaginal malignancies can be used in staging, but the tumor, node, metastases (TNM) staging system appears to be more widely used in case reports (2, 3, 8). At presentation, over half of the cases are localized or locally advanced, which is consistent with our patient’s case (5, 9). Despite the lack of a standard staging system for vulvovaginal melanomas, certain clinicopathologic features were found to be prognostic. These include a worse prognosis estimated for a deeper Breslow thickness (primarily for vulvar melanoma) (4), regional lymph node involvement, worse TNM stage, tumor size ≥ 3 cm (for vaginal melanomas) (10), and older age (1, 5, 11, 12). Our patient had locally advanced disease with a tumor size that exceeded 3 cm that by MRI imaging appeared to extend to the right parametrial wall and possibly involved the anterior rectal wall. There was no apparent regional lymph node involvement.

In terms of survival outcomes for patients with vaginal melanomas, these have estimated to range from 5 to 30%, even though most cases are first diagnosed without evidence of distant metastatic disease (1, 10, 13, 14). A recent U.S. population-based analysis reported a median overall survival for patients with vaginal melanomas of 16 months and the disease-specific survival of 19 months (9). This is significantly worse when compared to cutaneous melanoma at any stage. For our patient, at the time of submission of this report continued to be without evidence of disease recurrence by radiologic and gynecologic examinations at about 26 months of follow up from the time of immunotherapy initiation.

Mucosal melanomas have a low incidence of BRAF mutations at around 6% with mutations in KIT and NRAS estimated at 13% and 8%, respectively (15, 16). Therefore, molecularly targeted therapy has very limited implications in the treatment of mucosal melanoma. Hou et al. found c-KIT mutations in 22% of 51 vulvovaginal melanomas (37 vulvar and 14 vaginal) versus 3% of 2127 cutaneous melanomas (p< 0.001). It was also noted that vaginal melanomas specifically had a higher incidence of NRAS mutations compared to vulvar melanomas. This was also demonstrated by Omholt et al., where NRAS mutations were found in 43% (3/7) of vaginal melanomas (16), which is the case of our patient’s tumor. These different molecular differences between vulvar and vaginal melanomas suggest that, despite anatomic proximity, they probably develop through distinct pathogenetic mechanisms. Furthermore, PD-1 (67%) and PD-L1 (25%) were found to be commonly expressed in vaginal melanomas (15).

Surgical resection has been considered the mainstay of therapy and the only option for a potential cure. Surgeries ranging from wide local excision to pelvic exenteration have been used (8). Compared to surgical resection in cutaneous melanomas, extensive pelvic surgical procedures lead to loss of urinary continence and sexual function, and in younger patients, loss of reproductive ability. Multiple retrospective analyses have failed to show a survival difference between conservative and more extensive surgical resections. In a retrospective review by Miner et al. for vaginal melanomas, median recurrence-free survival was 12 months compared to10 months (P = 0.53), respectively, for radical versus wide local excision (5, 8). Sentinel lymph node mapping with completion lymphadenectomy is often considered for vulvar melanomas but its role in vaginal melanomas is unclear mostly due to the extensive lymphatic drainage (17). Primary local radiation can be an alternative option but has been shown to yield inferior results compared to surgery (8, 12), although it can be used in the adjuvant setting or at the time of local disease recurrence (18). For our patient, definitive surgical management would have involved posterior pelvic exenteration that the patient declined. The implemented immunotherapeutic approach resulting in complete tumor resolution and organ preservation provided the most optimal outcome in this case.

There have been no clinical trials dedicated to vulvovaginal melanomas that evaluated systemic therapies in the adjuvant or metastatic setting. However, multiple studies tested immunotherapy in patients with metastatic mucosal melanoma. Neither traditional dacarbazine-based chemotherapy nor biologic therapy with interferon have been shown to be effective (6). A case series of 4 patients with unresectable mucosal melanoma (3 vaginal, 1 cervical) at Memorial Sloan Kettering were treated with front-line ipilimumab followed by radiation (6). Three of the four patients underwent surgical resection and one achieved complete pathologic response. Two of the patients were reported to have a durable complete remission (6). A pooled analysis of 86 metastatic mucosal melanoma patients treated on nivolumab and nivolumab/ipilimumab in clinical trials showed that combination immunotherapy produced a median progression-free survival of 5.9 months and objective response rates of 37.1%. A prospective analysis of checkpoint inhibitors in advanced mucosal melanomas (including 27.3% with vulvovaginal disease) showed an objective response rate (ORR) of 8.2% (one complete response) for ipilimumab and 35% (four complete responses) for pembrolizumab. In a subgroup analysis of the phase 3 CheckMate-067 trial in patients with metastatic inoperable mucosal melanoma, the ORR with nivolumab plus ipilimumab in mucosal melanoma was 43% compared to 30% for nivolumab and 7% for ipilimumab. The 5-year overall survival rates were 36%, 17% and 7%, respectively (19).

Targeted therapies have also been evaluated in mucosal melanoma. Imatinib has been used in cutaneous and mucosal melanoma patients with KIT mutations with limited activity reported (19–21). BRAF inhibitors are likely of less utility given the lower percentage of BRAF mutations in mucosal melanomas. Our patient’s melanoma had an NRAS mutation where MEK inhibitors may have a role as systemic therapy, although a modest and short-lived responses are generally expected based on the experience with metastatic cutaneous melanoma. Furthermore, MET aberrations have been frequently demonstrated in melanoma and an interplay between MET and PD-L1 expression has been reported in cutaneous and mucosal melanoma (22), supporting experimental strategies involving combinatorial approaches of MET inhibition and immunotherapy that may have a role in this disease (23).

We observed enhanced cytotoxic T-cell infiltration and increased expression of PD-L1 and MHC-I in the tumor following immunotherapy. Studies in cutaneous melanoma with neoadjuvant ipilimumab and nivolumab + ipilimumab reported enhanced immune infiltration in responding tumors (24, 25). Enhanced MHC-I expression further potentiates the immunotherapeutic response where impaired MHC-I is known to negatively impact T-cell recognition and activation, and compromise antitumor immunity (26). Therefore, enhanced MHC-I expression on tumor cells facilitates their immune recognition while increased PD-L1 expression makes the tumor an ideal target for anti-PD1 immunotherapy.

To our knowledge, our case is the only published report of a patient with locally advanced vaginal mucosal melanoma experiencing a durable complete pathologic response and organ preservation following immune checkpoint blockade with ipilimumab and nivolumab as the only treatment approach. This case supports future research into neoadjuvant immunotherapeutic strategies to treat locally advanced vaginal mucosal melanoma under multidisciplinary care, early on-treatment assessment for response and close monitoring for immune mediated adverse events (27).

## Patient perspective

During my treatment for melanoma, I received three cycles of ipilimumab and nivolumab. The main symptoms that I experienced were minor and included skin rash, mild bloating and cough. Overall, I was able to maintain an active lifestyle without significant disruptions. Unlike other forms of treatment, such as chemotherapy and radiation, I did not experience any drastic changes or a decrease in my energy level and appetite. More importantly, I was able to maintain my dignity as a woman. I could not accept pelvic exenteration as the first treatment option for my case. The treatment plan for my rare advanced mucosal melanoma involving immunotherapy has resulted in the best outcome we envisioned.

## Data availability statement

The raw data supporting the conclusions of this article will be made available by the authors, without undue reservation.

## Ethics statement

Ethical review and approval were not required for the study on human participants in accordance with the local legislation and institutional requirements. The patients/participants provided their written informed consent to participate in this study. Written informed consent was obtained from the individual(s) for the publication of any potentially identifiable images or data included in this article.

## Author contributions

All authors contributed to the write up of the manuscript and approved the submission.

## Acknowledgments

We would like to thank our patient for allowing us to be part of the clinical care team.

## Conflict of interest

AT reports grants contracted with the institution from Bristol Myers Squib, Genentech-Roche, Regeneron, Sanofi-Genzyme, Nektar, Clinigen, Merck, Acrotech, Pfizer, Checkmate, OncoSec. Consulting fees from Bristol Myers Squibb, Merck, Easai, Instil Bio, Clinigen, Regeneron, Sanofi-Genzyme, Novartis, Partner Therapeutics, Genentech/Roche, BioNTech. All outside the submitted work.

The remaining authors declare that the research was conducted in the absence of any commercial or financial relationships that could be construed as a potential conflict of interest.

## Publisher’s note

All claims expressed in this article are solely those of the authors and do not necessarily represent those of their affiliated organizations, or those of the publisher, the editors and the reviewers. Any product that may be evaluated in this article, or claim that may be made by its manufacturer, is not guaranteed or endorsed by the publisher.

## References

[1] Sanchez A Rodríguez D Allard CB Bechis SK Sullivan RJ Boeke CE . Primary genitourinary melanoma: Epidemiology and disease-specific survival in a large population-based cohort. Urol Oncol: Semin Orig Investig (2016) 34(4):166.e7–166.e14. doi: 10.1016/j.urolonc.2015.11.009 26739672

[2] Kalampokas E Kalampokas T Damaskos C . Primary vaginal melanoma, a rare and aggressive entity. a case report and review of the literature. In Vivo (2017) 31(1):133–9. doi: 10.21873/invivo.11036 PMC5354139 28064232

[3] Jamaer E Liang Z Stagg B . Primary malignant melanoma of the vagina. BMJ Case Rep (2020) 13(1): e232200. doi: 10.1136/bcr-2019-232200 PMC7021152 31996380

[4] Piura B . Management of primary melanoma of the female urogenital tract. Lancet Oncol (2008) 9(10):973–81. doi: 10.1016/s1470-2045(08)70254-7 19071254

[5] DeMatos P Tyler D Seigler HF . Mucosal melanoma of the female genitalia: a clinicopathologic study of forty-three cases at duke university medical center. Surgery (1998) 124(1):38–48. doi: 10.1016/S0039-6060(98)70073-X 9663250

[6] Schiavone MB Broach V Shoushtari AN Carvajal RD Alektiar K Kollmeier MA . Combined immunotherapy and radiation for treatment of mucosal melanomas of the lower genital tract. Gynecol Oncol Rep (2016) 16:42–6. doi: 10.1016/j.gore.2016.04.001 PMC4899413 27331137

[7] Payne KK Mine JA Biswas S Chaurio RA Perales-Puchalt A Anadon CM . BTN3A1 governs antitumor responses by coordinating alphabeta and gammadelta T cells. Science (2020) 369(6506):942–9. doi: 10.1126/science.aay2767 PMC7646930 32820120

[8] Miner TJ Delgado R Zeisler J Busam K Alektiar K Barakat R . Primary vaginal melanoma: a critical analysis of therapy. Ann Surg Oncol (2004) 11(1):34–9. doi: 10.1007/bf02524343 14699031

[9] Wohlmuth C Wohlmuth-Wieser I May T Vicus D Gien LT Laframboise S . Malignant melanoma of the vulva and vagina: A US population-based study of 1863 patients. Am J Clin Dermatol (2020) 21(2):285–95. doi: 10.1007/s40257-019-00487-x PMC7125071 31784896

[10] Buchanan DJ Schlaerth J Kurosaki T . Primary vaginal melanoma: thirteen-year disease-free survival after wide local excision and review of recent literature. Am J Obstet Gynecol (1998) 178(6):1177–84. doi: 10.1016/s0002-9378(98)70320-5 9662299

[11] Lotem M Anteby S Peretz T Ingber A Avinoach I Prus D . Mucosal melanoma of the female genital tract is a multifocal disorder. Gynecol Oncol (2003) 88(1):45–50. doi: 10.1006/gyno.2002.6848 12504626

[12] Petru E Nagele F Czerwenka K Graf AH Lax S Bauer M . Primary malignant melanoma of the vagina: long-term remission following radiation therapy. Gynecol Oncol (1998) 70(1):23–6. doi: 10.1006/gyno.1998.4982 9698468

[13] Ragnarsson-Olding B Johansson H Rutqvist LE Ringborg U . Malignant melanoma of the vulva and vagina. trends in incidence, age distribution, and long-term survival among 245 consecutive cases in Sweden 1960-1984. Cancer (1993) 71(5):1893–7. doi: 10.1002/1097-0142(19930301)71:5<1893::aid-cncr2820710528>3.0.co;2-7 8448754

[14] Chang AE Karnell LH Menck HR . The national cancer data base report on cutaneous and noncutaneous melanoma: a summary of 84,836 cases from the past decade. the American college of surgeons commission on cancer and the American cancer society. Cancer (1998) 83(8):1664–78. doi: 10.1002/(sici)1097-0142(19981015)83:8<1664::aid-cncr23>3.0.co;2-g 9781962

[15] Nassar KW Tan AC . The mutational landscape of mucosal melanoma. Semin Cancer Biol (2020) 61:139–48. doi: 10.1016/j.semcancer.2019.09.013 PMC7078020 31655118

[16] Hou JY Baptiste C Hombalegowda RB Tergas AI Feldman R Jones NL . Vulvar and vaginal melanoma: A unique subclass of mucosal melanoma based on a comprehensive molecular analysis of 51 cases compared with 2253 cases of nongynecologic melanoma. Cancer (2017) 123(8):1333–44. doi: 10.1002/cncr.30473 28026870

[17] Omholt K Grafström E Kanter-Lewensohn L Hansson J Ragnarsson-Olding BK . KIT pathway alterations in mucosal melanomas of the vulva and other sites. Clin Cancer Res (2011) 17(12):3933–42. doi: 10.1158/1078-0432.Ccr-10-2917 21680547

[18] Leitao MM Jr . Management of vulvar and vaginal melanomas: current and future strategies. Am Soc Clin Oncol Educ Book (2014), e277–81. doi: 10.14694/EdBook_AM.2014.34.e277 24857113

[19] Shoushtari AN Wagstaff J Ascierto PA Butler MO Lao CD Marquez-Rodas I . CheckMate 067: Long-term outcomes in patients with mucosal melanoma. J Clin Oncol (2020) 38(15_suppl):10019–9. doi: 10.1200/JCO.2020.38.15_suppl.10019

[20] Komatsu-Fujii T Nomura M Otsuka A Ishida Y Doi K Matsumoto S . Response to imatinib in vaginal melanoma with KIT p.Val559Gly mutation previously treated with nivolumab, pembrolizumab and ipilimumab. J Dermatol (2019) 46(6):e203–4. doi: 10.1111/1346-8138.14763 30614559

[21] Hodi FS Corless CL Giobbie-Hurder A Fletcher JA Zhu M Marino-Enriquez A . Imatinib for melanomas harboring mutationally activated or amplified KIT arising on mucosal, acral, and chronically sun-damaged skin. J Clin Oncol (2013) 31(26):3182–90. doi: 10.1200/jco.2012.47.7836 PMC4878082 23775962

[22] Song KY Desar S Pengo T Shanley R Giubellino A . Correlation of MET and PD-L1 expression in malignant melanoma. Cancers (2020) 12(7): 1847. doi: 10.3390/cancers12071847 32659961 PMC7408820

[23] Sun ZJ Wu Y Hou WH Wang YX Yuan QY Wang HJ . A novel bispecific c-MET/PD-1 antibody with therapeutic potential in solid cancer. Oncotarget (2017) 8(17):29067–79. doi: 10.18632/oncotarget.16173 PMC5438713 28404966

[24] Amaria RN Reddy SM Tawbi HA Davies MA Ross MI Glitza IC . Neoadjuvant immune checkpoint blockade in high-risk resectable melanoma. Nat Med (2018) 24(11):1649–54. doi: 10.1038/s41591-018-0197-1 PMC6481682 30297909

[25] Tarhini AA Edington H Butterfield LH Lin Y Shuai Y Tawbi H . Immune monitoring of the circulation and the tumor microenvironment in patients with regionally advanced melanoma receiving neoadjuvant ipilimumab. PloS One (2014) 9(2):e87705. doi: 10.1371/journal.pone.0087705 24498358 PMC3912016

[26] Gu SS Zhang W Wang X Jiang P Traugh N Li Z . Therapeutically increasing MHC-I expression potentiates immune checkpoint blockade. Cancer Discovery (2021) 11(6):1524–41. doi: 10.1158/2159-8290.CD-20-0812 PMC8543117 33589424

[27] Tarhini AA Eads JR Moore KN Tatard-Leitman V Wright J Forde PM . Neoadjuvant immunotherapy of locoregionally advanced solid tumors. J Immunother Cancer (2022) 10(8):e005036. doi: 10.1136/jitc-2022-005036 35973745 PMC9386211

